# Personal

**Published:** 1902-03

**Authors:** 


### PERSONAL.

Dr. Edwin Ricketts, of Cincinnati, was a recent visitor in Buffalo, the guest of Dr. William Warren Potter. He was delighted with the fine sleighing that afforded an opportunity for a ride on the snow, which proved a rare treat to him.

Dr. and Mrs. Charles G. Stockton, of Buffalo, sailed February 8, 1902, on the Celtic for the Mediteranean trip, to be absent until the middle of April.

Dr. W. J. O'Donnell, of Buffalo, formerly house surgeon at the Emergency Hospital, whose office is at No. 269 Carolina Street, has been appointed physician to the Buffalo Natural Gas and Fuel Company.

Dr. L. H. Laidley, of St. Louis, has been appointed medical director of the World's Fair, to be held in that city in 1903.

Dr. A. L. Benedict, of Buffalo, has removed from 998 Main Street to The Roanoke, 156 W. Chippewa Street, corner Morgan Street. Practice limited to diseases of the digestive organs: stomach, intestine, liver. Hours: 8 to 9, 2 to 3, 6.30 to 7.30; Sundays, 12 m. Telephone, Tupper 670. At 795 Ellicott Square, 5 to 6 p.m.

Dr. Walter L. Savage, of Buffalo, Niagara University, 1897, has been appointed medical examiner of immigrants at this port. That position is connected with the United States Marine Hospital Service. Dr. Savage was informed recently that he had passed the federal civil service examination for the place and he has assumed the duties of the office.

---

Dr. Edward A. Southall, U. of B. '98, a member of the Board of Health, Manila, P. I., has been appointed to the medical examining and licensing board of the Philippine Islands.

---

Dr. Henry L. Elsner, of Syracuse, retiring president of the Medical Society of the State of New York, read a paper before the Buffalo Academy of Medicine, medical section, Tuesday, February 11, 1902, as announced elsewhere. He was entertained at dinner by Dr. Julius Ullman, who invited about a dozen Buffalo physicians to meet the distinguished ex-president.

---

Dr. Mary Clayton, of Buffalo, passed the recent civil service examination for state hospital appointments at the head of the list.

---

Dr. and Mrs. Edward T. Smith, of Buffalo, are spending a few weeks in the south, and their trip includes a visit to Charleston and St. Augustine.

